# Impacts of Climate Change on Outdoor Workers and Their Safety: Some Research Priorities

**DOI:** 10.3390/ijerph16183458

**Published:** 2019-09-17

**Authors:** Haruna M. Moda, Walter Leal Filho, Aprajita Minhas

**Affiliations:** 1 Department of Health Professions, Manchester Metropolitan University, Manchester M16 6BG, UK; H.Moda@mmu.ac.uk; 2 Research and Transfer Centre “Sustainable Development and Climate Change Management”, Hamburg University of Applied Sciences, 21033 Hamburg, Germany; Aprajita.minhas@haw-hamburg.de

**Keywords:** adaptation strategy, outdoor workers, heat stress, research, developing countries

## Abstract

The literature on the potential impacts of climate change on the health of outdoor workers has received limited attention as a whole, and in sub-Saharan African countries in particular. Yet, substantial numbers of workers are experiencing the health effects of elevated temperature, in combination with changes in precipitation patterns, climate extremes and the effects of air pollution, which have a potential impact on their safety and wellbeing. With increased temperatures within urban settlements and frequent heats waves, there has been a sudden rise in the occurrence of heat-related illness leading to higher levels of mortality, as well as other adverse health impacts. This paper discusses the impacts of extreme heat exposure and health concerns among outdoor workers, and the resultant impacts on their productivity and occupational safety in tropical developing countries with a focus on Sub-Saharan Africa, where there is a dearth of such studies. Aside from the direct effects caused by extreme heat exposure, other indirect health hazards associated with increasing heat among this group includes exposures to hazardous chemicals and other vector-borne diseases. In addition, reduced work capacity in heat-exposed jobs will continue to rise and hinder economic and social development in such countries. There is an urgent need for further studies around the health and economic impacts of climate change in the workplace, especially in tropical developing countries, which may guide the implementation of the measures needed to address the problem.

## 1. Introduction

Global climate change is among the most visible environmental concerns of the 21st century and these changes have the potential to affect human health, both directly and indirectly. Urban centers in most developing countries are now witnessing rapid population growth. According to the United Nations, the world’s urban population is expected to increase to about 57% by 2050. Developing countries will account for more than 90% of future population growth experienced within its cities. With this projection of population growth, the WHO has urged its member states to take decisive action aimed at addressing the health impacts associated with climate change [1]. Despite being one of the most recognized contemporary and future global environmental issues, climate change impacts and it’s adverse aspects to human lives, including occupational safety, have received surprisingly little attention [2]. Because of the rapid global urbanization trend, urban heat island (UHI) phenomena are now part of the climatological effects resulting from human activities on the urban environment [3].

Kiefer et al., argued that, despite the existence of considerable research and planning with regard to the public health and environmental aspects of climate change, there is little effort focused on its effects on workers’ health and safety [4]. Workers, especially those working outdoors, are often the first to be exposed to the effects of climate change. They may be exposed for longer durations and at greater intensities which in the long run could result in the increase in prevalence and severity of known occupational hazards and exposures, and also the emergence of new ones. Previous research has shown that climate change can contribute to a decrease in the ozone layer and affect UV radiation levels at the surface of the earth. This can cause outdoor workers to experience more frequent, intense, and longer exposure to UV radiation, resulting in an increased risk of adverse eye effects, skin cancer, and possibly immune dysfunction [4,5,6]. In addition, exposure to higher temperatures with more frequent periods of heat may result in greater heat stress, potentially leading to more cases of heat-related illnesses such as heat stroke, heat exhaustion, increased susceptibility to chemical exposure, and fatigue. Exposure to increased temperature can also result in reduced vigilance creating an increased risk of injury or lapses in safety. Furthermore, elevated temperatures can increase levels of air pollution, including ground-level ozone; outdoor workers have longer exposure to such air pollutants, which are linked to chronic health effects, such as respiratory diseases and allergic reaction [7,8,9,10,11,12].

The report compiled by TUC [13] has acknowledged that climate changes are expected to bring about both risks and opportunities to every sector of the country’s economy. In the energy sector, for instance, the direct impact of climate change could result in power plant flooding, leading to power cuts affecting other economic sectors and on the demand side, energy use for indoor cooling during the summer is expected to increase. Workers required to respond to problems that may arise could be placed at higher risk during these extreme weather events. Seven categories of climate-related hazards—increased ambient temperature, air pollution, ultraviolet exposure, extreme weather, vector-borne diseases and expanded habitats, industrial transitions and emerging industries and changes in the built environment at work—have been the subject of climate change assessments on the health of outdoor workers in several studies [4,8,9,10,11,12]. There has been an increasing concern around the impact of extreme heat on both indoors and outdoors workers health and safety due to increased heat and climate change [14,15,16,17]. As pointed out by St. Louis and Hess [18] the health impact of climate change will not be distributed uniformly, but rather it is expected that the distribution patterns of health burdens will be increased around the globe.

The purpose of the paper is to summarize the existing knowledge and synthesize the impact of climate change adaptation and occupational health and safety. In order to achieve this, the paper provides concise a review concerning new findings around relevant health impacts associated with climate change, extreme heat exposure and comments on different adaptation strategies available to decision makers to alleviate the impact of climate change factors and outdoor workers’ productivity. Due to limited research work carried out in tropical developing countries, especially in sub-Saharan Africa, the paper aims to draw more research attention around emerging research areas as they relate to the impact of extreme heat exposure, climate change adaptation measures and health and safety concerns among outdoor workers (individuals that spend more than 4 h working outside) within these countries. It will consider the likelihood of effects on their productivity and occupational safety. Aside from the direct impact caused by extreme heat exposure, other indirect health hazards associated with increasing heat among this group include exposure to hazardous chemicals and other vector diseases, which will also be addressed. By proposing micro-adaptation alternatives, this study will help policymakers adopt effective means of meeting the challenges posed by climate change exposure.

## 2. Materials and Methods

A narrative review of previously published literature was undertaken to generate data that support the development of the work. A broad perspective on the subject was taken in the review, that helps to describe the impacts of climate change, adaptation and occupational health and safety concerns among outdoor workers.

### 2.1. Search Strategy and Sources of Information

**Figure 1:**
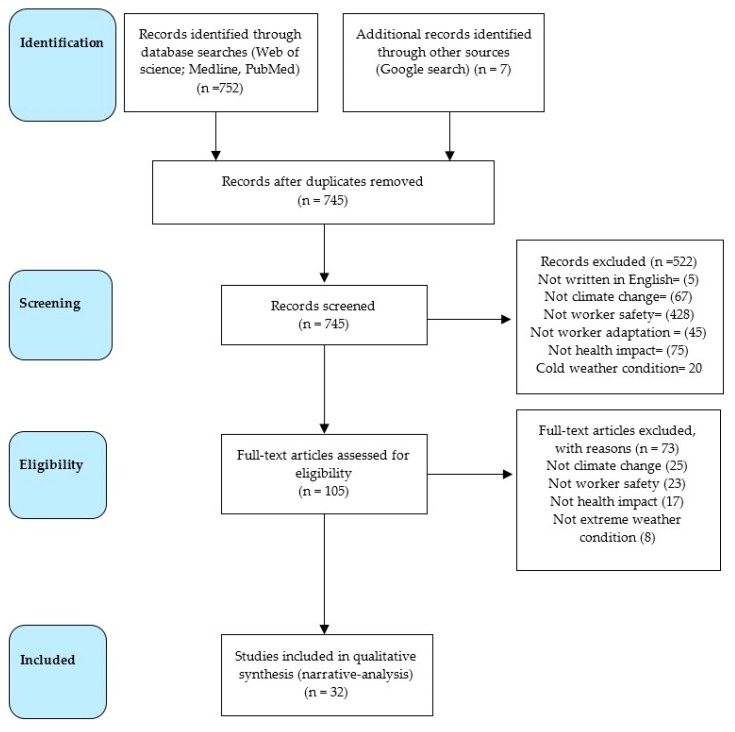
Adopted PRISMA flowchart providing insights as to how the studies were selected [19].

The study adopted the Preferred Reporting Items for Systematic Reviews and Meta-Analyses (PRISMA) guidelines to identify relevant materials for inclusion in the study [19]. To identify relevant peer reviewed articles and grey documents we searched the Google Scholar, PubMed, Medline and Web of Science databases from January 2002 to March 2019 for studies that assessed the impact of climate change on workers’ health and productivity. In addition, electronic databases searches were supplemented with manual searches for relevant published studies on the subject on international agencies’ websites that include the WHO, UNDP, IPPC and ILO. Based on the considered criteria 32 studies were included in the present study from among the initial 745 ones identified earlier (Figure 1).

Keywords used during each search included ‘climate change impact’ and; ‘urban heat island’, ‘extreme heat’, ‘heat strain and health’ heat stress and productivity’, ‘outdoor workers health’, ‘occupational health and safety’, ‘health and safety’, ‘adaptation’. Only articles that meet the needs of the present paper regarding climate change impacts on workers safety (hazards, risk) and health (disease, chemical exposure, and zoonosis) were considered. No restrictions were applied on articles that reported on workers’ health status in relation to the study aim. Because of the target research area, sub-Saharan Africa, articles that considered extreme cold weather conditions were excluded. In addition, studies on the subject but published in any language other than English were excluded. Further exclusion criteria considered are presented in Table 1.

### 2.2. Characteristics of the Considered Studies

The characteristics of the studies here reviewed include ten entries for each: article type, study design, data sources, analysis method, study aim; study population, study theme, intervention, outcome data and outcome measures. Table 2 provides a detailed overview of the 32 studies considered in the review. Geographically the studies varied widely across the countries of the continents of Africa, Asia, Europe, North and Central America and Australia, so as to ensure a wide international basis, hence adding robustness to the findings. The designs varied among the studies and included empirical evidence, systematic reviews; scenario-based assessments, narrative reviews; exploratory studies; survey-based studies; formative research, case studies and ecological study design. Other measures considered in the appraisal of selected studies included reporting style, outcome measures, study design, the fidelity of protocol and possible conflicts of interest.

## 3. Results and Discussion

Based on the period selected (2002–2019) for the included studies, 22 (68.8%) of them were published between 2014 and 2019. Of the 32 papers analyzed, four papers (12.5%) directly considered the impacts of climate change on workers’ health in different countries (Ghana, South Africa, Saudi Arabia, Germany, Australia, USA, Italy, India). Four (12.5%) of the papers considered the impacts of climate change, workplace heat exposure and heat stress among female workers. Seven (21.9%) of the papers focused on climate change, workplace heat exposure, heat stress, etc. within the construction industry. Overall, the trends of topics identified from the papers considered include the influence of climate change and heat stress, workplace injury and work productivity.

From the data abstraction undertaken based on keywords adopted; 32 studies selected were grouped into five major themes. This was also done based on their similarities, emerging patterns and differences. The themes that emerged, based on the study categories, include:

(a) Climate change impacts on outdoor workers safety and health;
(b) Urban Heat Island (UHI) and Occupational Health Impacts on Outdoor Workers;
(c) Heat stress and outdoor workers performance;
(d) Occupational health hazards and effects related to climate change;
(e) Adaptation of workers to occupational heat stress.

Among the included studies, the impacts of climate change on workers’ health was the major commonality while their major differences were around the interventions considered. Broad findings from the studies revealed that exposure to extreme heat due to climate change is associated with negative health impacts and possible decreases in productivity. In addition, the need for sentinel effects and leading indicators to aid surveillance of climate related occupational effects have also been highlighted in several of the studies. Different study designs were adopted among the studies considered, while a mixed method approach was adopted as the analysis method by 44% of the studies.

### 3.1. Climate Change Impacts on Outdoor Workers’ Safety and Health

At the turn of the century, urban areas (especially in underdeveloped and developing countries such as those in sub-Saharan Africa) witnessed a sudden surge of movement from rural settlements into urban areas in search of better living conditions. This trend has resulted in greater pressures to the urban environment, especially considering that around 40% of the population in the African continent are now living in and around urban settlements, as reported by the World Bank. In addition, the migration process has been supported by the diversity of economic and social opportunities available in these urban settlements within the African Continent [48,49]. Temperatures across the African continent are expected to increase faster than the global average, while mean annual precipitation is projected to decrease in and around the Mediterranean and Northern and Southern parts of the continent. However, precipitation in the Western part of the continent will vary. Further to this, the near surface temperatures in most parts of Africa has seen an increased temperature rise of 0.5 °C or more in the last century, with minimum temperatures warming more rapidly than maximum temperatures. These trends may have been influenced by other components of natural variability and human activity [50].

To understand the impacts caused by climate change, there is first the need to understand the phenomenon itself. The availability of manmade heat absorbing features such as concrete buildings, surface modification, pollution generated from automobiles etc. in urban areas has helped increase heat absorption during the day, and its gradual release back into the atmosphere at nighttime. This has had an impact on temperature differences between urban and rural areas, and sped up the urban heat island (UHI) phenomenon in urban areas [48,49]. Another contributory factor to the UHI effect is the absence of moisture in urban areas and increased anthropogenic heating [31]. This increase has been shown to have direct significant effects on outdoor workers’ thermal comfort, higher energy usage and air quality, with detrimental health effects and possible mortality increase [49]. The indirect impacts resulting from unsustainable consumption, such as pollution increase and waste generation, are also seen in these areas [44]. In addition, changes in land use and land cover in urban areas has influenced the urban climate and is leading to an increase in temperature [31]. There is now a need for evidence-based studies on climate change adaptation and urban heat island effects in relation to outdoor workers. This can raise awareness of occupational health hazards in order to establish risk awareness and coping strategies among workers, managers, and other stakeholders [25,27,32,38]. While there is anecdotal evidence based on research carried out in other parts of the world regarding excessive heat exposure and its impact on workers’ health, safety, productivity and workplace environmental conditions and adaptation strategies, there is a paucity of similar data among outdoor workers in parts of sub-Saharan Africa; especially in a changing climate as perceived today [14,25,29,32,36].

### 3.2. Urban Heat Island (UHI) and Occupational Health Impacts on Outdoor Workers

An urban heat island (UHI) is an urban area that exhibits higher temperatures compared to the rural or suburban surroundings. The UHI effect is due to various factors, such as air pollution, anthropogenic heat, urban architecture and variations in precipitation patterns [31,48]. The UHI impacts on human health through the exposure to increased temperatures and can be problematic specifically during heat waves [48]. While the UHI effect affects the public in general, there is also the need for attention to the effects it poses on the health of workers specifically. Heat exposure has been previously linked to various adverse health effects, from the aggravation of minor conditions such as general discomfort, heat cramps, respiratory difficulties, heat stroke to increased chances of hospitalization and even death [4,5,6,25,49].

Health impacts from UHI are more severe during the summertime, the season of immensely high temperature or heat waves. Thus, heat-related mortality is likely to increase in future due to climate change [31,51]. Heat mortality may occur due to the overloading of the cardiovascular and respiratory systems, as physiological reactions to heat exposure. The physiological reactions that take place are increased heart rate, increased body temperature, increased sweating, fluctuation of blood flow towards the skin from the central organs, and dehydration [52]. The Urban Heat Island is also a nocturnal phenomenon, resulting in increased temperature at night due to the release of heat. This increased temperature causes a lack of relief at night and prevents the body from recovering from the heat exposure experienced during the day [53].

The UHI phenomenon can lead to an increase in energy consumption due to the demand for more use of cooling devices, thus increasing the overall electricity use. The companies which supply the electricity rely on power plants which typically use fossil fuels to meet the required demand, which results in the release of air pollutants and emission of greenhouse gases. These gases include Sulphur dioxide, nitrogen oxides, carbon monoxide, among others, all of which negatively impact air quality and contribute to ground-level ozone formation, particulate matter generation and acid rain. The elderly population, minors and those with existing heart conditions are most likely to be affected by these effects [51,54]. Increases in wind speed may help to reduce the severity of [24], but only to a limited extent.

Along with climate change, the impact of heat in the urban area will increase in the future. People working in hot weather involving heavy physical activity without appropriate protection are at increased risk of suffering from heat-related health effects [1,55]. In the urban inner cities, the major effect of UHI is human discomfort which is well documented in previous urban heat stress studies [56]. The UHI effect increases the temperature in cities exposing the urban population to more heat stress compared to rural areas [43]. In August 2003, during two weeks of extreme heat more than 1000 deaths and several associated illnesses of people aged 20–70 occurred in France [55] which greatly impacted the nations working group.

The ‘heat island effect’ is partly responsible for the current changes in temperature in many cities. People working outdoors (such as traffic wardens, fire fighters, road sweepers, landscapers’ petty traders, construction workers etc.), during the hottest periods face an additional 3–5 °C increase in temperature which will make heavy work very challenging [40]. When working in a hot environment, workers (including healthy ones) are under tremendous strain. Their sweating (body’s cooling mechanism) efficacy is reduced due to limited air movement towards the skin. In addition, the protective clothing used by workers which protects them from exposure to chemicals, trauma, and other pollutants, may hinder evaporative heat loss, further reducing the sweating efficacy. Another factor that may increase UHI is the inadequate intake of water which results in dehydration, and therefore reduces sweating and resultant heat loss. Dehydration also contributes to impairment of mental and physical performance [25,27,42].

There is a strong relationship between the UHI effect and urban planning. This is due to the fact that the absence of trees and vegetation in urban areas affects the transpiration process. The implementation of proper vegetation in urban areas helps cool the surroundings, resulting in increased evapotranspiration and reducing the UHI effect. Other measures include the use of water bodies to reduce thermal load and decreasing the anthropogenic heat [44]. Table 3 lists some examples of health impacts of UHI on outdoor workers.

### 3.3. Heat Stress and the Performance of Outdoor Workers

While individuals are in general capable of acclimatizing to different levels of heat, it is worth noting that every worker also has an upper limit to heat exposure, stress beyond which will become unbearable and may cause health related ailments and—in extreme cases—can lead to mortality. Understandably, there is limited, or no adaptation measures considered for outdoor workers involved with constant physical labor and working in humid conditions. There is an increased likelihood of these workers experiencing heat stress, which could lead to reduced work performance and capacity, with potentially significant economic consequences [39]. Wherever the ambient temperature exceeds 35 °C, there is a greater chance of it causing fatigue and physical exhaustion among workers in general. There is also an increased risk of errors, which could be catastrophic, especially where concentration is required for the safe handling and operation of machinery. Outdoor women worker’s health is another problem that has drawn attention recently; in particular, during pregnancy as it creates additional heat stress problems. Respiratory and cardiovascular disease, secondary to exposure to poor air quality, has been found to have a larger impact on women because of their greater propensity for higher deposition of particulate matter in lung tissue [17,33,59]. In general, outdoor workers are faced with elevated risks of heat-related illnesses (HRI). However, pregnant women exposed to extreme heat are faced with additional health risks, including poor pregnancy health and birth outcomes, as highlighted in earlier reports [17,26,33,34,59]. This calls for empowering women through provision of education and awareness as a means of improving mitigation and mitigation policy intervention.

As earlier mentioned by Kjellstrom et al., there are two pathways that extreme heat exposure impacts could manifest in workers [36]. Both physiological and psychological tasks and heat balance within the human body are determined by six fundamental factors that include; air temperature; radiant temperature; humidity; air movement (wind speed); clothing; and the metabolic heat generated by human physical activity. As such, perceptual awareness alongside the control over work conditions, work rates, and work limits are immediate adaptation mechanisms against heat exposure among individual workers. To address the physiological impact caused by heat exposure, several heat stress indices have been developed. These have been developed in order to help in the prediction of physiological strain experienced among workers due to exposure to stressful environmental conditions [15,28,29,35,36]. The use of indexes provides information between climate parameters (air temperature, air humidity, air movement over the skin (wind speed) and heat radiation (i.e., from the sun)) which can then be linked to a corresponding physiological strain. The results of these indices can be used in the design or establishment of safe work practices, work limits and work conditions. Wet Bulb Globe Temperature (WBGT) was developed by the US Army in the 1950s to help reduce exposure to excess heat. WBGT is now the most widely used tool for occupational heat stress assessment. It takes into consideration four environmental factors; air temperature, relative humidity, wind speed, and radiation [30,60]. However, clothing type, activity and acclimatization can significantly impact on the adaptation strategy adopted by the individual, despite the use of WBGT to quantify heat stress tolerance [29,61]. As such, the interpretation of the WBGT value requires these factors to be taken into account.

Guidelines for the application of WBGT on occupational heat exposure recommend maximum heat exposures for jobs with various work intensities. They also need to account for the number of work hours after which a worker will be required to take a break to avoid the core body temperature exceeding 37 °C [40]. Reference to WBGT ‘reference values’, (the point at which some preventive action should be taken) as developed by the international standard [62], provide further guidance on the different levels of work where workers will need hourly rests, or rests of 25, 50 and 75% (rest/work ratios).

### 3.4. Occupational Health Hazards and Effects Related to Climate Change

There are links between prevailing climatic factors and occupational health hazards that can be associated to climate change. Likely hazards due to climate change and their effects on vulnerable outdoor groups, as well as aliments associated with occupational exposure to excessive heat have been identified. These include; heat-associated self-reported nausea or vomiting, painful muscular spasms, confusion, dizziness, or fainting, hot dry skin and self-reported heat strain according to previous studies [6,25,36].

Outdoor workers in certain occupational sectors such as agriculture, construction, transportation, utility maintenance, oil production, firefighting and other emergency services are usually among the first to experience the effects of climate change. These are exacerbated in most instances by the need to wear protective clothing, which can lead to heat stress. In addition, such effects could have a far-reaching impact on their health and the nation’s economy. Excessive environmental heat is seen as the most frequent climate change impact and as such, with prolonged hot weather, outdoor workers health in sub-Saharan African countries, are at risk from heat-related outcomes (Table 1).

Where these workers are exposed to a higher temperature than 37 °C, for their body to stay at a healthy temperature they will have to lose this excessive heat through sweat evaporation. However, as earlier reported [59] high external air humidity and clothing type were identified as limitations sweat evaporation and regulation of body temperature. Therefore, to avoid heat stroke workers will need to reduce their work rate, take more rest, and rehydrate. However, Moda and Alshahrani [25] reported that outdoor workers on construction sites in Saudi Arabia described the lack of palatable water as a key set back to their water intake and rehydration. This is often caused by the portable water provision on site becoming warm during the day due to lack of a cooler, thus leaving the workers dehydrated, exhausted and at some point, experiencing severe fatigue among other symptoms reported. In addition, heat stress and fatigue suffered by these workers negatively impacted their levels of alertness and work capacity. This led to the frequent occurrence of safety lapses leading to a high risk of injury at work. Most workplaces in developing countries do not have a heat stress index on site, therefore workers rely on environmental temperature references from the weather station for the city, as reported by Moda and Alshahrani [25]. Unfortunately, the immediate local ambient temperature could vary from that reported by the weather station and the workers might be exposed to a more extreme temperature than reported.

Apart from the effects of extreme heat, a higher temperature can lead to increased ground-level ozone and fine particulate matter air pollution, thereby increasing the risk of cardiopulmonary dysfunction, reduced lung function and other respiratory illnesses (Table 3). These other effects also increase the level of carbon dioxide in the air and promote plant growth and the release of airborne allergens, which could increase allergic reaction and asthma episodes among vulnerable groups [63,64,65]. To help visualize climate change impacts and related occupational safety and health issues, Schulte and Chun [5] developed a conceptual framework. This framework highlights the impact climate change is likely to have on the workplace among different workers, including occupational morbidity, mortality, and injury as influenced by several driving and contextual factors. The likely hazards that will occur in different settings may include increased ambient temperature; air pollution; ultraviolet (UV) radiation; extreme weather; expanded vector habitats; industrial transitions and emerging industries; and changes in the built environment.

Release and exposure to environmental chemicals due to increased heat is expected to increase through various routes. These include increased pesticide use, changes in transport pathways such as dust proliferation, increased chemical dispersal from storm runoff, and increases in chemical spills from floods and fires [20]. Several workers in trades or industries using or producing petroleum products (such as coal etc.), and those working in close proximity where the combustion of these products occur (such as traffic wardens, taxi/bus drivers, road maintenance etc.) are vulnerable to polycyclic aromatic hydrocarbon (PAH) exposure. However, their impact due to climate change are said to vary. In addition to this, the presence and exposure of legacy pollutants (persistent substances like DDT, dioxins, PCBs, mercury, etc.) that have accumulated in environmental reservoirs (such as surface soils, sediments, and forests), especially among workers in certain occupations (Table 4), could be influenced by climate change [20,21]. These pollutants impact health, including, but not limited to, cancer, adverse reproductive outcomes, impaired neurodevelopment, and disruption of the endocrine and immune systems [21].

In addition, workers in areas with poor water drainage management and areas that encourage vector breeding are affected by extreme climate events. This is especially concerning considering that some of these vector borne diseases i.e., yellow fever, malaria, dengue and chikungunya are sensitive to climatic changes and likely to expand in geographic zones and affect a diverse range of worker populations [10,11,66]. The burdens associated with vector borne diseases tend to be much higher in developing countries. For instance, the per capita mortality rate from vector-borne diseases is projected to be 300 times greater in developing nations than in developed regions. This is due in part to their prevalence in the tropical regions and low socioeconomic development, which has a negative impact on the quality of health care services delivered [10].

### 3.5. Adaptation of Workers to Occupational Heat Stress: Some Research Priorities

There is the need for occupational climate change policies be considered at a micro level, especially since adaptive capacity may vary between groups, communities, and individuals and will rely on the vulnerability level, resilience, and resource availability as the global temperature continues to rise. Heat stress exposure and associated health effects cannot be ignored in the workplace, especially in sub-Saharan Africa where climate change is more pronounced. On this note, it is imperative that climate change adaptation be considered at various workplace levels and is not a collectivist approach, which does not take into account the diverse needs of the varying workers.

The intergovernmental Panel on Climate Change (IPPC) affirmed that if the present climate change trend persists, by the middle of the century, high temperatures and humidity would probably compromise outdoor working. Thus, leading to lost work capacity and reduced labor productivity among vulnerable populations, such as that in sub-Saharan Africa, and will eventually cause economic loss. To avoid productivity and economic loss and social ramifications resulting from extreme heat exposure among outdoor workers, there is a need for employers to consider measures capable of protecting workers and their businesses through investment into appropriate climate change adaptation measures [12,59]. Historically, individuals working under extreme climate have tried different measures aimed at adapting to their work conditions. They include, working with light cloth, dousing themselves with water to regulate their internal body temperature, consumption of water, taking intermittent breaks etc., however, these techniques require further scientific measures aimed at complementing these tools, especially where it is insufficient for coping with extreme hot weather conditions.

Key research priorities need to meet this challenge. It is firstly important to investigate effective adaptation measures to ensure workers involved in heavy labour are not faced with an increased risk of heat stress, which could affect their health, work performance and work capacity [39]. Further to this, there is also a need to consider the right adaptation strategy among workers when developing occupational guidelines. These guides should take into account several factors likely to play a role in climate change and heat exposure, which could affect the individual’s sensitivity to heat tolerance [5]. In addition, there is also a need to consider intervention strategies around workers coping mechanisms, including adaptation and social protection measures when designing engineering solutions. Furthermore, research on the role of administrative controls along with studies on how to enhance education and awareness on the management of climate change and heat exposure among workers in general is considered timely, especially in Africa. There is also the need to ensure the establishment of a platform capable of overcoming barriers to climate change adaptation and heat stress risk which takes into consideration resource availability and technological advancements in tropical regions [38].

## 4. Conclusions

From the studies considered, it is evident that the frequency and intensity of extreme hot weather conditions due to climate change, extreme workplace heat exposure and the abatement of workplace ill health and injury will continue to present challenges, in the developing countries located in the tropics, and the globe at large, especially in fast-paced work environments. To date, most studies on climate change impacts and health have focused on the general public’s health. Limited documented evidence exists on climate change impact on occupational health and safety among outdoor workers, especially in developing countries in sub-Saharan Africa where its impact is mostly felt. While approaches are considered for the protection of workers from extreme heat exposure due to climate change, there is also the need for the development of appropriate surveillance programmes, thus enabling the proper assessment of occupational heat exposure and related injury and illness because of climate change extremes within different occupational sectors.

As a result of paucity of up to date data on outdoor workplace heat exposure, workplace climate change adaptation strategies and other relevant related health and safety issues, the assessment of impacts of climate change and how it affects workers in the tropical countries such as Africa is considered timely. This includes the need for current information on the effective management of climate change impacts. In addition, gender response to climate change impacts on the continent workforce will require the development of more evidence, where a high proportion of women now work in fast-paced outdoor industries that include agriculture, mining and construction. To strengthen the knowledge of climate change, workplace heat exposure and related workplace injury and to guide safety adaptation measures, there is a need for multidisciplinary research to help in the quantification and forecast of workers hazards exposure by occupation and location in these countries where the impact of climate change is more pronounced. Research areas proposed in the assessment of climate change impacts on outdoor workers productivity and occupational safety include:

- Assessment of climate change impacts among vulnerable outdoor workers; such as pregnant women, children elderly in the continent
- Occupational heat stress and adaptation of sustainable measures
- Association between heat stress exposure and response under varied working conditions
- Assessment of combined effects associated with heat stress and other related environmental and physical stressors
- Development of micro adaptation alternatives to tackle workplace climate change challenges

To measure the occupational illness and injury burden among outdoor workers, research that considers the relationship between socioeconomic status and other relevant indicators that affect climate change, and occupational safety and health of these workers is further advocated for. Another area of study that needs looking at is the interplay between climate change, occupational hazards, and workers vulnerability. This is needed in order to help with the development of suitable climate change adaptation and risk management initiatives.

To conclude, climate change policy needs to take into consideration that at each micro level adaptive capacity may vary between groups, communities, and individuals and will rely on the vulnerability level, resilience, and resource availability. As such, there is a need to avoid a collectivist approach, as this approach does not acknowledge these differences.

## Figures and Tables

**Table 1:** Inclusion and exclusion criteria considered in the selection of studies.

Inclusion Criteria	Exclusion Criteria
Study outcome that considers either quantitative, qualitative and mixed-method approaches	Comments, letters, editorials, viewpoints, reviews, reports, and correspondence
Peer-reviewed journal articles published in English language	Articled that are published in other languages
Studies on climate change impact on workers’ health and safety, heat stress and heat strain, adaptation strategies	Climate change-related studies that places emphasis only on storms, rainfall, drought, cyclones, and rising sea levels
Studies that considered the impact of urban heat island effect on workers’ health and wellbeing	Articles that are not related to the context of the study
Assessment of occupational heat stress on psychological and social well-being	Studies only on the effect of climate change and heat stress on plants, animals, and crops
Studies on barriers of workers to occupational heat stress adaptation	Studies using only secondary data without primary data

**Table 2:** Characteristics of the materials included in the review.

Author and Year	Study Design	Analysis Methods	Study Aim	Study Populations	Study Theme	Study Intervention	Outcome Data	Outcome Measure
Applebaum et al. [20]	Empirical evidence	Mixed methods	Overview on exposure to climate change elements and vulnerable occupational sectors	US workers	Occupational risk and climate change impacts	Climate change threats to workers	Anticipation of how workers will be susceptible to climate change impacts	National research agenda around control and mitigation of workers susceptibility to climate change
Flouris et al. [6]	Systematic review	Mixed methods	Develop policies and programmes aimed at assessing climate change impacts on health, economics and social benefit	Varied	Environmental heat strain and worker productivity and health effects	Review on occupation heat strain on workers’ health and productivity	Single shift workers working under heat stress are more likely to experience occupational heat strain	Actions to mitigate climate change effects and anticipated rise in heat stress
Balbus et al. [21]	Scenario based assessments	Not stated	Association between global climate change, chemical spread and risk to human health	Varied	Global climate change and the use and spread of chemical in the environment	Influence of global climate change on the exposure to chemicals and their resultant health impacts	Review of policies to address global climate change influence on chemical risk	Generation of Improved data set to determine human exposure to chemical matrix associated with climate change variability
Xiang et al. [22]	Cross-sectional survey	quantitative method	Investigation of workers’ extreme heat exposure perceptions and behavioural responses in warm climate	Varied	Climate change, heat stress, workplace heat exposure, and work-related injuries	Impact of climate change and workers’ perceptions and attitudes towards workplace heat exposure	The need to strengthen workers’ heat risk awareness and refine current heat prevention strategies in a warming climate	Promotion of educational programmes and training among varied workforce especially the those with limited education level
Messeri et al. [23]	Case study	Mixed methods	Assessment of impact of culture on heat-stress perception and management among native and immigrant workers	Migrant workers	Migrant occupational risk, heat waves and heat perception	Assessment of perceive high temperature risk in workplace among different worker force	Ethnical differences on heat stress perception and adaptation strategies	Not stated
Mathee et al. [14]	Pilot study	Mixed methods	Assessment of outdoor workers perceptions of hot weather and the impacts on their health and productivity levels	Varied	Climate change, global warming impact on outdoor workers health and productivity	Assessment of potentials workforce populations current adaptation measures to cope with hot weather	Poor coping ability in very hot conditions, and difficulty maintaining work output during very hot weather	Call to improve workers knowledge on extreme heat exposure and its health effect and health promotion strategies
Lundgren et al. [24]	Systematic review (Pearl picking)	Mixed methods	Assessment on the effects of heat stress on working populations	Developing countries	Climate change impact and occupational heat strain on workers	Review of information climate change effect on vulnerable groups in developing countries	Urban heat island effect, physical work, individual difference can exacerbate heat stress on workers	Adoption of preventive and control measures to achieve multiple benefits
Moda and Alshahrani, [25]	Survey-based quantitative case study	Univariate and bivariate analysis	Assessment of the relationship between temperatures and heat morbidity among outdoor workers	Outdoor construction workers, Jizan	Extreme heat exposure and adaptation strategy	Assessment of adaptation strategy on workers response to extreme heat and its health impact	High rate of heat-induced injuries and illnesses and decrease of work productivity	Update on policy development around occupational heat stress and its risks within the region
Sett and Sahu, [26]	Scenario based assessments	Univariate and bivariate analysis	Evaluation of workplace heat exposure, and productivity of female brickfield workers	Female brickfield workers-West Bengal, India	Heat exposure; and cardiac strain, workload; productivity	Heat stress exposure and work productivity impact	Encourage ergonomic interventions, rescheduling of the work rest cycle, frequent fluid intake	Not stated
Schulte and Chun, [5]	Systematic review	Mixed methods	Development of a framework for the identification of climate change impact on work place, workers and occupational morbidity and mortality	Working populations	Climate change effects and associated occupational hazards	Relationship between exposure to occupational hazards and incidence of morbidity, mortality and injury related to climate change impact	The use of sentinel effects and leading indicators to aid surveillance of climate related occupational effects	Conceptual framework developed to aid decision makers to assess occupational health policy and recommendations
Morioka et al. [27]	Cross-sectional study	Mixed method	Assessment of hot working environment at construction site in summer and health effect on workers	Workers on site	Hot environment, outdoor work condition and associated health effect	Measurement of blood urea nitrogen, blood sugar, serum osmotic pressure and associated health effect	Preventive heat-stress measures such as adequate ventilation, palatable water and taking needed rest needed to reduce heat stress	Adaptation of administrative control-health education and training
Crowe et al. [28]	Qualitative evaluation	Exploratory interviews	Assessment of heat-related health issues within the sugarcane industry	Sugarcane workers-	Climate change impact, heat stress, heat exposure and agriculture	Strategies for reducing heat-related health effects and impact measurement of workers productivities	Promote better understanding of the multiple factors drivers for the improvement of workers’ health and safety	Not stated
Lucas et al. [29]	Scenario based assessments	Not mentioned	Asses present and future ergonomic risk associated with working in extreme heat	Varied	Climate change, heat stress and occupational injury	Management of heat strain and reduce risk of serious ill health	Mandatory protection to occupational heat to reduce impact of excessive heat exposure	Ensure clothing properties and thermoregulation are understood and managed appropriately
Hancock and Vasmatzidis, [30]	Formative research	Not mentioned	Review of current knowledge state around the effect of heat stress on cognitive performance	Varied	Impact of heat stress on cognitive performance	Assessment of appropriate heat stress index to measure heat stress intensity in relation to cognitive work	The use of factors i.e., age gender, level of experience, motivation and training to better understand impact of heat stress on cognitive performance	Not stated
Heaviside et al. [31]	Scenario based assessments	Mixed methods	Review of health impacts associated with urban heat island through heat exposure	Varied	Estimations of the impacts of various climate change mitigation techniques and benefits to workers health and wellbeing	Quantitative estimate on UHI health impacts and measurement of UHI health mitigation measures	The need to highlight associated health risk climate change mitigation measures adaptation	Not stated
Numfan et al. [32]	Case study	Mixed methods	Assess perception of climate change and occupational heat stress and adaptation strategies among mining workers	Supervisory personnel in government and private sectors, Ghana	Climate change perception and risk of occupational heat stress and adaptation strategies	Role of supervisors in the implementation of occupational heat stress mitigation among mining workers	Association between workers level of education and willingness to adopt control measures to mitigate against occupation heat stress linked to climate change	Development of awareness and training on heat stress management among mining workers
Sorensen et al. [33]	Narrative	Not mentioned	Case for polices to move beyond traditional separations and advancement of gender-based solution	Varied	Integration of gender-based awareness in climate change intervention strategies	Plans and policies shift to reduce gender-based bias in the implementation of climate change adaptation strategies	Women’s health outcome and economic prosperity as surrogate markers for policies and projects aimed at reduction in disaster risk and climate change adaptation	Improve reporting mechanism based on common indicators
Flocks et al. [34]	Community based participatory	Thematic analysis	Assess work practices, individual risk factors and physiological response of female workers in hot environments	Hispanic and Haitian nursery and fernery workers in Florida	Beliefs, perception and health related illness and pregnancy health	Awareness of heat related health effects on pregnancy and fetal health	Measure to better address heat as specific occupational hazard among women and pregnancy health	Not stated
Varghese et al. [35]	Systematic review	Thematic analysis	Assessment of the relationship between heat exposure and occupational injuries	Varied	Climate change, health and safety and workplace heat exposure	Impact of workplace heat exposure and occupational injuries	The need for an increased awareness of injury risk during hot weather and the economic benefits associated with averting injury, poor health outcomes and lost productivity	Investigate specific injuries and the workers at risk due to workplace heat exposure
Kjellstrom et al. [36]	Ecological design	Narrative	Assessment of special risk, health risk policies and strategies in the South-East Asia Region	South East –Asia regions	Threat to occupational health and productivity in South East Asia regions	Reduction of greenhouse gases from sources beyond current national plans	Adoption of effective prevention of workplace heat stress	National analysis and report on climate change impact
Acharya et al. [37]	Scoping review	Mixed methods	Assessment of the severity with which construction workers are affected by heat stress, risk factors and co-morbidities associated with heat-related illnesses	Varied	Climate change, heat related illness among construction workers and heat stress	Review heat-related Illnesses risk among construction workers	Knowledge gap around heat related health effects among construction workers despite the in global temperatures	Assessment of construction workers exposure–response associations under a large range of temperatures and across locations and development of effective intervention and prevention action plans
Numfan et al. [38]	Systematic review	Mixed methods	Review of climate change risks and heat stress exposure on employees health and safety, productivity and social well-being	Varied	Awareness of occupational heat stress, social impacts and adaptation strategies	Workers awareness and adaptation strategy to occupational heat stress exposure	Adaptation strategies key for policy development aim at improving occupational heat stress	Improve policies around occupational stress management
Kjellstrom et al. [39]	Scenario based assessments	Mixed methods approach	Assessment of physiological as indicative measures of reduced wok capacity and human performance due to heat increase	Indoor and outdoor workforce in tropical and subtropical regions	Review of Climate change impact, productivity and socioeconomic impact	Prevention of clinical damage to organs function and diminished human performance capacity due to climate change	Social and economic impact due to climate change	Climate change related occupational health impact assessment
Kjellstrom et al. [7]	Case series	Triangulation method	Assessment of climate change extent on labour productivity due to increased temperature and humidity under future projections	Workforce in different world region and climate types	Assessment of Climate change impact and labour productivity	Climate change adaptation measure and its impact on human systems	Adaptation measures between high income nation and low income counterpart may varies	Climate change impacts and adaptation strategies at local and country level
Kjellstrom et al. [40]	Scenario based assessments	Formative methods	Introduction of occupational heat stress index and how workers are likely to be affected by different level of heat exposure	Workers in low and middle income tropical countries	Heat exposure impact on productivity and occupational health	Adaptation measures in low and middle income countries outdoor workers	Need for effective preventive measure to reduce occupational heat stress and reduce the burden on socioeconomic development	Adoption of appropriate preventive measures in planning process for work environment and urban development
Al-Bouwarthan et al. [41]	Scenario based assessments	Mixed methods	Assessment of work factors related to heat stress exposure among construction workers	Construction workers–Saudi Arabia	Extreme heat exposure, climate change impact and construction workers	Development of occupational heat exposure guidelines	Call for assessment of both short and long term health impacts due to prolong heat exposure	Develop threshold based on heat index/WBGT for heat stress risk at work
Hanna et al. [42]	Scenario based assessments	Not mentioned	Examine the emerging risk for working people and review of national occupational health and safety policy	Working population	Heat exposure and adaption	Review of public health policy	Health risk associated with heat exposure among workers	Climate change adaptation and Occupational health and safety guidelines
Ylipaa et al. [17]	Qualitative research design	Not mentioned	Assessment of impacts and vulnerabilities of workers to climate change	Varied: Agriculture workers	Climate change adaptation; gender inequality; feminist political ecology	The need for inclusive and Situation-Based climate change adaptation policies	Gender-informed climate change adaptation that acknowledges important conditions	Call for recognition of social relations and location, in national strategies and policies to support national targets on climate response, gender equality, and sustainable development
Ward et al. [43]	Case study	Spatial comparison, Univariate and bivariate analysis	Investigate causes of surface urban heat island and impact of urban pattern and land use characteristics	European cities with different climate zone and population density	Surface heat island magnitude and heat waves	Introduction of heat magnitude to assess added heat load during heat waves	Case specific adaptation strategies for urban planning	Development of heat stress risk evaluation measures
Leal Filho et al. [44]	Narrative review	Mixed method	To improve the knowledge basis of urban heat islands and the scale of vulnerability	Two regions: Germany and Australia	Urban heat island vulnerability cities and climate change mitigation and adaptation	Mitigating the impacts of urban heat islands	Increased vulnerability of cities to the negative impacts of urban heat	Urban heat island mitigation and adaptation strategies which take the particularities of each community into account
Xiang et al. [45]	Systematic review	Thematic analysis	Review of workplace heat exposure characteristics in high risk occupations	Varied: farmers, construction workers, fire-fighters, miners, soldiers, and manufacturing workers	Work related injury, heat exposure and climate change	Effect of workplace heat exposure due to climate change and work related injury	Potential impacts of workplace heat exposure are underestimated as a result of underreporting of heat related illnesses	Not stated
Chersich and Wright, [46]	Systematic review	Thematic analysis	Assessment of climate change adaptation policy frameworks and review of preparedness levels and action around extreme weather events	South African health sector	Climate change adaptation, extreme weather, health systems, health policy	Climate change adaptation policy and extreme weather preparedness level	Climate change adaptation policy status and level preparedness against extreme conditions	Effective use of data, strengthening of the health profession, increased health sector leadership
Sheng et al. [47]	Case-crossover study	Stratified case-crossover method	Association between high temperature exposure and work related injury	Working groups, Guangzhou	Relationship between extreme temperature and work related injury	Associated injury risk at work due to hot weather conditions	Estimation of future impacts of climate change on workers and adaptation strategies	Not stated

**Table 3:** Health Impacts of Urban Heat Island (UHI) on Outdoor Workers.

Health Impacts	Author & Year
Heat exposure Heat Stress/Stroke Fatigue Dehydration and Kidney Disease Cardiovascular Disease Respiratory Distress Death Increase morbidity and fatality	Leal Filho et al. [48] Ward et al. [43] Kjellstrom et al. [40] Heaviside et al. [31] Hanna et al. [42] Tan et al. [57] Kovats and Hajat, [56]
Air Pollution Respiratory Distress Respiratory Track Irritation Asthma Attack Increased Respiration due to Heat exposure Exposure to carcinogens	Kjellstrom et al. [40] Ward et al. [43] Kjellstrom et al. [52]
Unbalanced Physiological Function leading to decrease in work capacity	Lucas et al. [29] Lundgren et al. [24] Kjellstrom et al. [40] Kovats and Hajat, [56]
Extreme weather and sea level rise High risk of flooding causing displacement Injury Resource disruption e.g., water supply	Kjellstrom et al. [52] McGranahan et al. [58]
Psychological effects on Workers Mental health	Kjellstrom et al. [7] Lundgren et al. [24] Hanna et al. [42] Kjellstrom et al. [52]

**Table 4:** Potential impact of climate change on occupational sectors by exposure source.

Contaminant Type	Occupation at Risk	Exposure Route	Health Effect	Reason for Likely Increase
Pesticides	Agriculture, landscape	Dermal, inhalation, ingestion	Numerous: carcinogenic, Asthma, COPD, cardiopulmonary etc.	Increase in plant disease
Veterinary medicines	Veterinary, agriculture	Dermal, ingestion	Antimicrobial resistance	Increased temperature
Ozone	Construction, transportation, energy, agriculture, traffic warden, oil and gas etc.	Inhalation	Asthma, COPD, cardiopulmonary	Increased temperature
PAHs	Construction, transportation, energy, agriculture, traffic warden, oil and gas, firefighting etc.	Inhalation	Cardiopulmonary, carcinogenic	Increased dust, forest fires
Pathogenic microorganism	Fishing, agriculture, sanitation, most outdoor work	Dermal, Inhalation, ingestion	Infectious disease	Increased flooding, soil and water contamination
Vector-borne infectious agents	Food-animal production, most outdoor work	Dermal	Infectious disease	Increased range of vectors
Soil dust	Agriculture, construction, most outdoor work	Inhalation, ingestion	Silicosis, cardiopulmonary	Drier conditions
Industrial processing chemicals	Chemical manufacture, emergency response operations	Dermal, Inhalation, ingestion	Numerous: Carcinogenic, Asthma, COPD, cardiopulmonary etc.	Flood, wildfires
Wildfire smoke	Firefighting, agriculture,	Inhalation	Respiratory	Drier conditions
Exposure to extreme condition (temperature and humidity)	Firefighting, oil and gas workers, and all outdoor workers exposed to direct sun,	Dermal, Inhalation, Ingestion	Heat exhaustion, heat stroke, chronic kidney disease, chemical poisoning, injury	Extreme condition
Other indirect climate-related hazards	Low-income groups with limited health protection; workers with existing non-climate health problems affected by heat	Dermal, inhalation, Ingestion, other	Infectious diseases, non-communicable diseases, mental health issues, etc.	Others

Adapted and modified from Applebaum et al. [20] and Kjellstrom et al. [36].
